# Spectroscopic Ellipsometry Study on Tuning the Electrical and Optical Properties of Zr-Doped ZnO Thin Films Grown by Atomic Layer Deposition

**DOI:** 10.1021/acsaelm.1c01026

**Published:** 2022-02-24

**Authors:** Carolina Bohórquez, Hicham Bakkali, Juan J. Delgado, Eduardo Blanco, Manuel Herrera, Manuel Domínguez

**Affiliations:** † Centro de Investigación Científica y de Educación Superior de Ensenada (CICESE), 22860 Ensenada, Baja California, Mexico; ‡ Departamento de Física de la Materia Condensada, Universidad de Cádiz, Campus de Puerto Real, E11519 Puerto Real, Spain; § Institute of Research on Electron Microscopy and Materials (IMEYMAT), Universidad de Cádiz, Campus de Puerto Real, E11519 Puerto Real, Spain; ∥ Departamento Ciencias de los Materiales e Ingeniería Metalúrgica y Química Inorgánica, Universidad de Cádiz, Campus de Puerto Real, E11519 Puerto Real, Spain; ⊥ Centro de Nanociencias y Nanotecnología, Universidad Nacional Autónoma de México, 22860 Ensenada, Baja California, Mexico

**Keywords:** Zr-doped ZnO, ALD, spectroscopic ellipsometry, optical properties, free carrier density, Drude−TL model

## Abstract

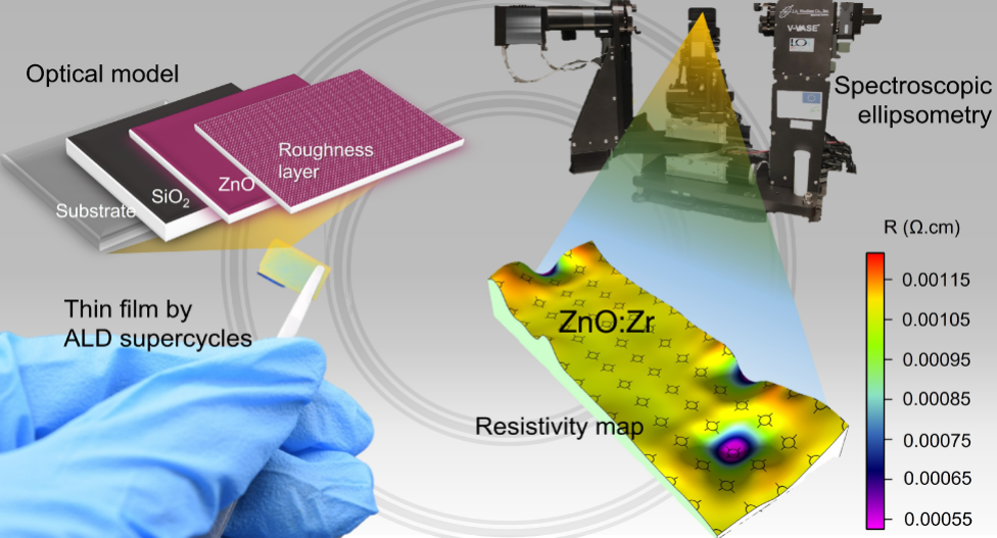

This work reports the ellipsometry analysis of atomic layer deposition (ALD) films of ZnO doped with Zr to determine parameters like free carrier concentration and mobility. Thin films of zinc oxide (ZnO) and Zr-doped ZnO of thickness ∼100 nm were prepared by atomic layer deposition on sapphire, SiO_2_/Si(100), and Si(100) substrates. Variable-angle spectroscopic ellipsometry was used to study their optical properties in the 0.5–3.5 eV spectral range. The optical constants were accurately obtained using a model that combines Drude and Tauc–Lorentz oscillators with Bruggeman effective medium approximations, allowing the inclusion of a roughness layer in the optical model. The effect of Zr doping (ca. 1.9–4.4 atom %) was then investigated in both as-prepared samples and samples annealed in the temperature range of 100–300 °C. All of the films exhibited good optical transparency (ca. 70–90% in the visible region). For doping levels below 2.7 atom %, the real part of the dielectric permittivity reveals a semiconductor-to-metal transition in the near-infrared (NIR) region, as the permittivity goes from positive to negative. Besides, the plasma energy increases with increasing Zr concentration, and both resistivity and carrier concentration exhibit slightly parabolic behaviors, with a minimum of ∼1.5 × 10^–3^ Ω cm and a maximum of 2.4 × 10^20^ cm^–3^, respectively, at the same critical Zr concentration (2.7 atom %). In contrast, the carrier mobility decreases rapidly from 76.0 to 19.2 cm^2^/(V s) with increasing Zr content, while conductivities and carrier mobilities worsen when the annealing temperature increases, probably due to the segregation of ZnO crystals. Finally, the optical band gap is very stable, revealing its interesting independence of substrate composition and annealing temperature, as it collapses to a single master curve when band gap energy is plotted versus free carrier concentration, following the Burstein–Moss effect. Overall, the Zr-doped ZnO films studied here would be a highly desirable system for developing thermally stable transparent conductive oxides (TCOs).

## 1 Introduction

Transparent conductive oxide (TCO) films such as indium tin oxide (ITO), tin oxide (SnO_2_), and zinc oxide (ZnO) are mainly used for electronic device manufacturing due to their singular optical transparency and high electrical conductivity, including liquid-crystal displays (LCD),^1,2^ flexible touch screens, organic light-emitting diodes (OLED),^3,4^ and photovoltaic devices.^5,6^ To date, ITO films have become the most commercialized ones, but they are limited by their high cost and poor surface roughness. As a promising substitute for ITO films, ZnO exhibits a hexagonal wurtzite crystal structure, relatively soft mechanical properties (with a hardness of 4.5 on the Mohs scale), and a direct and wide band gap of about 3.3 eV at room temperature. In addition, ZnO exhibits high chemical stability, good surface uniformity, and a large exciton binding energy of 60 meV. ZnO also has excellent electrical conductivity generated by its high concentration of donor native point defects, such as oxygen vacancies (V_O_) and zinc interstitials (Zn_i_), enhanced by doping with group IV elements including titanium,^7^ zirconium,^8^ hafnium,^9,10^ and ruthenium.^11^ Furthermore, Zr incorporation in ZnO lattice is chemically favorable due to the comparable ionic radii of Zn^2+^ and Zr^4+^ ions (74 and 73 pm, respectively) for the four-coordinate tetrahedral substitution, which generates two extra electrons by each ionic substitution.^12,13^ Consequently, Zr doping increases the electrical conductivity of ZnO at least until an overdoping limit is reached, from which the electrical resistivity, ρ, can increase or be limited, such as is reported for Al doping.^14,15^ Furthermore, Ellmer et al. previously reported that overdoping generates a critical limit for ρ of 2 × 10^–4^ Ω cm that remains constant. Besides, the mobility of free carriers did not overcome a value of 50 cm^2^/(V s).^16^ In general, this effect is a well-known property of TCO films,^17^ possibly due to the formation of dopant ions clusters near grain boundaries that increase carrier scattering.^100,18^^18^ On the other hand, the optical absorption of ZnO takes place throughout the superimposition of two separate absorption processes, attributed to the interband transitions in the near-UV region and to the free-electron transitions at the near-infrared (NIR) region.^19,20^ The first process is essentially governed by the band gap, which obeys the Burstein–Moss (BM) effect, while the plasma resonance of electron gas causes the second one in the conduction band. So far, ZnO optical response can be accurately modeled by combining Drude and Tauc–Lorentz (TL) oscillators, thus allowing the determination of all of the physically significant parameters involved in the absorption–conduction processes, such as free carrier density, mobility, concentration, resistivity, plasma frequency, and relaxation time.

In previous ellipsometric reports of ZnO films, the dielectric function and the optical constants *k* and *n* have been reported. For example, a significant parameter in the optical and electrical properties of TOC is thickness; a detailed study of this phenomenon was carried out by Samarasingha et al.^21^ They observe how the dielectric function changes in thin films with thicknesses from 5 to 50 nm (grown by atomic layer deposition (ALD)). This work used Tauc–Lorentz oscillators and simplified Herzinger–Johs oscillators; they also added a Gaussian oscillator in some thin films. The excitonic direct-gap peak is strongly broadened and weakened in thinner ZnO layers. In contrast, with fixed thickness, Fujiwara studied the electronic properties through ellipsometric spectroscopy in Ga-doped ZnO and ZnO thin films.^20^ They combined the Drude and Tauc–Lorentz oscillators to model the dielectric function. They found that by increasing the concentration of free electrons (through doping), there is a shift in ε_1_ from ∼3 eV toward higher energies. In ε_2_, the absorption increases at low energies due to these free electrons. In the case of Zr-doped ZnO thin films grown by ALD,^22−26^ ellipsometry has been used but only to determine the thickness of the films but not to determine optical or electronic parameters.

In this work, we appeal to a realistic model based on the Drude–TL combination in which a roughness layer, determined from atomic force microscopy (AFM) topography measurements, is also taken into account subsequently in the optical model as a Bruggeman effective medium approximation (EMA) layer.^27^ Thus, we first modeled the ZnO pure films and, second, the Zr-doped ZnO films deposited onto several substrates, both as-prepared and annealed at different temperatures ranging from 100 to 300 °C. The band gap evolutions, depending on these preparation conditions, were then studied. On the other hand, the critical limit of different parameters, such as free carrier concentration, resistivity, mobility, and relaxation time as a function of Zr doping level, was determined. We also show the potential of using the proposed optical model to map these parameters over a broad sample region as a function of film thicknesses. Finally, an example of a resistivity map is provided in this work.

## 2 Experimental Methods

**Figure 1:**
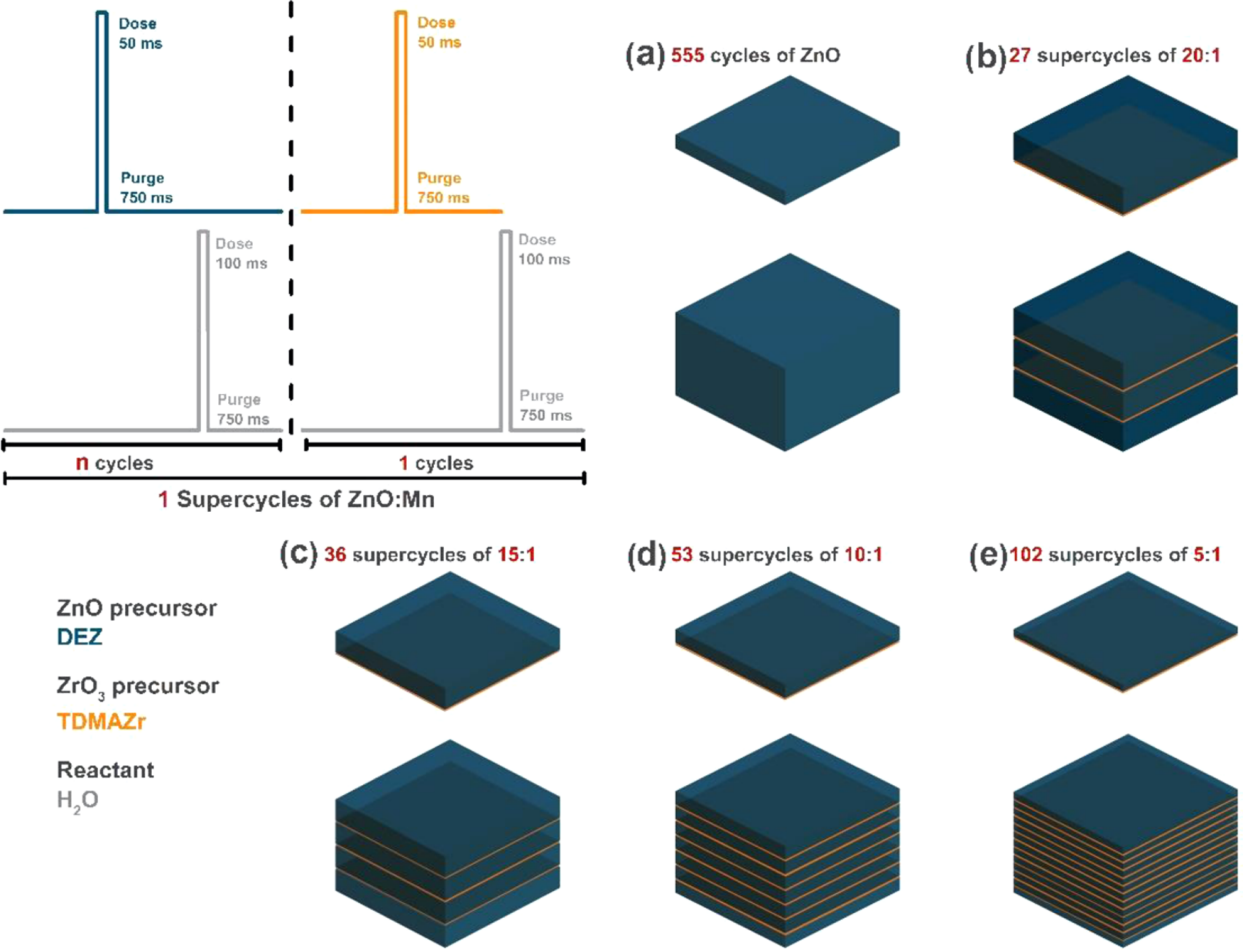
Scheme of the ALD cycle design. (Left) Conditions of a supercycle; (right) Number of supercycles for each doping concentration. (a) Reference ZnO and (b–e) different concentrations for 20–1, 15–1, 10–1, and 5–1 alternating supercycles, respectively.

Undoped ZnO and ZnO/Zr films were prepared onto sapphire(0001), Si(100), and SiO_2_/Si(100) substrates through a Beneq TFS ALD reactor.^25^ For the last substrates, the SiO_2_ layer was thermally grown on Si(100) at 1200 °C in an oxygen atmosphere until obtaining a thickness of 300 nm. The precursors for Zn and Zr were diethylzinc (DEZ; Zn [C_2_H_5_]_2_) (Strem 95%)^28^ and tetrakis(dimethylamido)-zirconium (TDMAZr; Zr[N[CH_3_]_2_]_4_) (Strem 98%), respectively. High-purity nitrogen (N_2_), with O_2_ traces below 10^–6^ ppm was used as a carrier gas to purge the growth chamber.^23^ Zr doping of ZnO films consisted of a defined number of supercycles composed of *n* cycles of ZnO by one cycle of Zr (labeled as *n*:1), with a growth rate of 1.8 Å for ZnO and 0.8 Å for Zr. Four Zr-doped ZnO films were prepared with cycle ratios of 20:1, 15:1, 10:1, and 5:1, applying 27, 36, 53, and 102 supercycles, respectively, to obtain similar thicknesses (Figure 1). An undoped ZnO film grown by 555 cycles was grown for reference. All samples were grown at a temperature of 200 °C. Subsequent thermal treatment at temperatures of 100, 150, 200, 250, and 300 °C for 1 h was performed on the films by applying a heat ramp with a rate of 5 °C/min in N_2_ as the carrier gas at a flow of 3 mL/min.

Phase, crystallite size, and lattice parameters of the films were characterized by X-ray diffraction (XRD) on a PANalytical X’Pert PRO MRD with a Cu Kα (λ = 1.540 Å) line excitation source in-plane diffraction conditions. The mean size of the crystallites, *D*, was calculated with the Scherrer equation
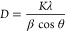
1where *K* is the Scherrer constant, λ is the X-ray wavelength, β is the full width at half-maximum (FWHM) of diffraction peaks, and θ is the Bragg angle. High-resolution (HREM) and scanning transmission electron microscopy (STEM) imaging were performed using a TEM/STEM FEI Talos F200X G2 microscope (Thermo Fisher Scientific, Waltham, MA) equipped with a high-angle annular dark-field (HAADF) detector, which was operated at 200 kV and with a camera length of 11.5 cm. X-ray energy-dispersive spectrometry (XEDS) mappings were performed using the 4 Super-X detector system integrated in the equipment. The beam current and the dwell time per pixel were 190 pA and 50 μs, respectively. To improve the visual quality of the elemental maps obtained, these were filtered using a Gaussian blur of 0.8 using Velox software. Phase identification was carried out using Eje Z software.^29^ The TEM specimen was prepared with a Thermo Scientific Helios Hydra DualBeam, which is a last-generation plasma focused ion beam scanning electron microscope (FIB-SEM). This equipment allowed us to prepare the ultrathin samples that are required for S/TEM studies. The film thicknesses and optical properties were determined by reflection through spectroscopic ellipsometry (SE). Before the ZnO synthesis on SiO_2_/Si(100) substrates, the thickness of the SiO_2_ layer was measured by SE analyzing the oxidized silicon substrates used to synthesize the ZnO films, with optical constants for c-Si and SiO_2_ provided with the WVASE software. SE spectra of Ψ and Δ were measured over a range of 0.5–3.5 eV with steps of 0.05 eV, at room temperature, using a Woollam v-VASE spectroscopic ellipsometer; it is a rotating analyzing ellipsometer (RAE) with a Berek computer-controlled adjustable MgF_2_ waveplate retarder (Automatic Retarder), which is used to accurately introduce a beam path delay over a wide spectral range. The variable retarder allows to adjust the input polarization to provide a reflected beam, which is always close to circularly polarized, and the system will measure Δ accurately over the entire range of 0–360°. Additionally, the autoretarder ellipsometer configuration permits the measurement of the % depolarization, which can be correlated with thickness nonuniformities of the samples, enabling a better fitting of the ellipsometric angles ψ and Δ in these cases. The data analysis was performed with the WVASE software from J.A. Woollam.^30^

To estimate the roughness layer thickness used in the optical model, the accumulated height distribution (a more sophisticated way of measuring the average crest–valley distance) was obtained from the atomic force microscopy (AFM) images of the undoped ZnO and ZnO/Zr films. We used a Bruker MultiMode NanoScope 8 AFM operated in tapping mode, using a SuperSharp SNL probe (nominal radius of curvature of the tip, 2 nm). The accumulated height distributions fitted the sigmoidal Boltzmann equation for each sample studied and introduced afterward in the optical model as a function-based effective medium approximation (EMA) layer.^27^ The optical transmission spectra were obtained using a UV–vis–NIR spectrophotometer from the thin films grown on sapphire.

## 3 Results and Discussion

### 3.1 Microstructural Characterization

**Figure 2:**
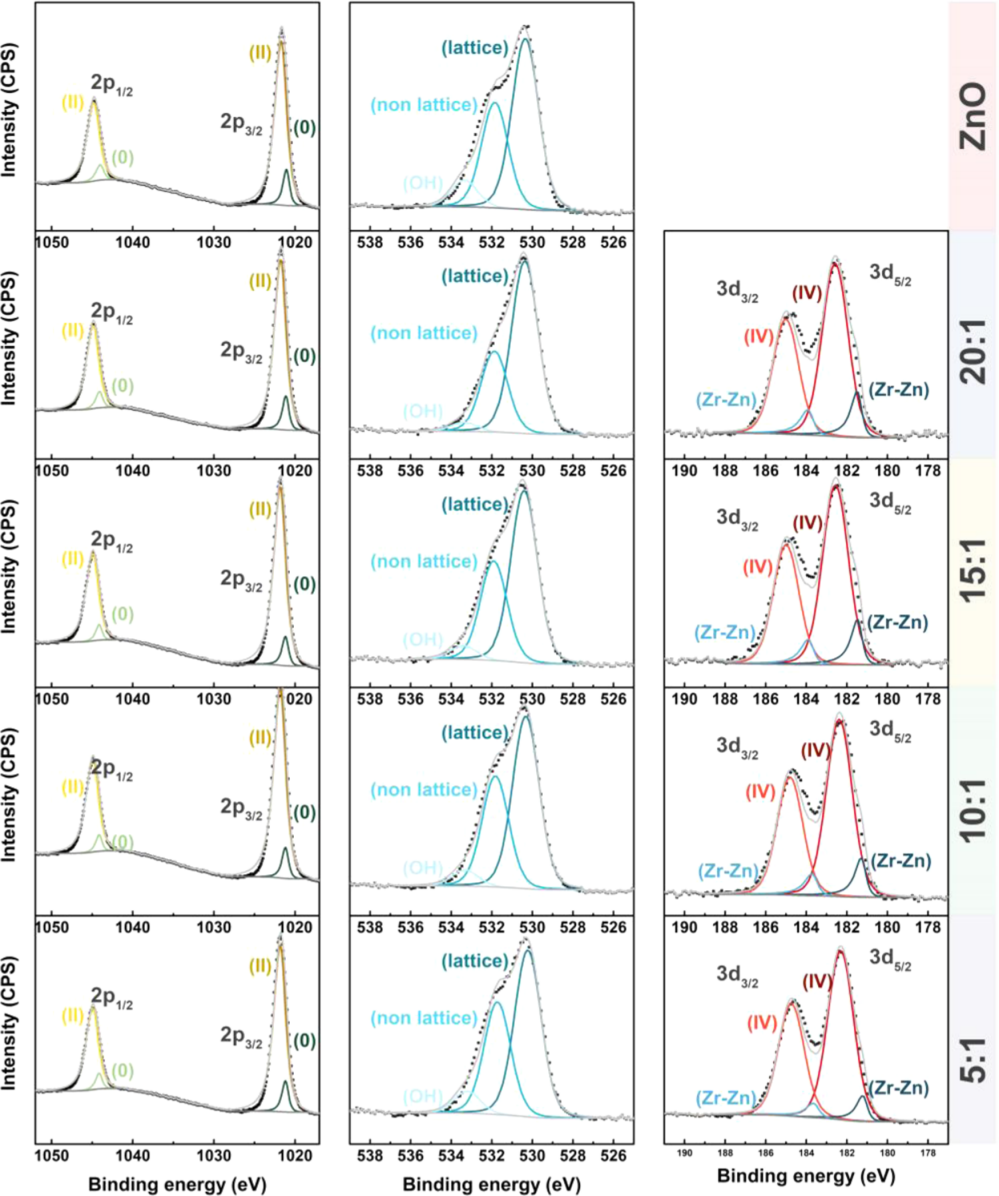
(Left) XPS spectra of the Zn 2p signals; (middle) O 1s and (right) Zr 3d spectra obtained from ZnO and ZnO/Zr samples.

**Figure 3:**
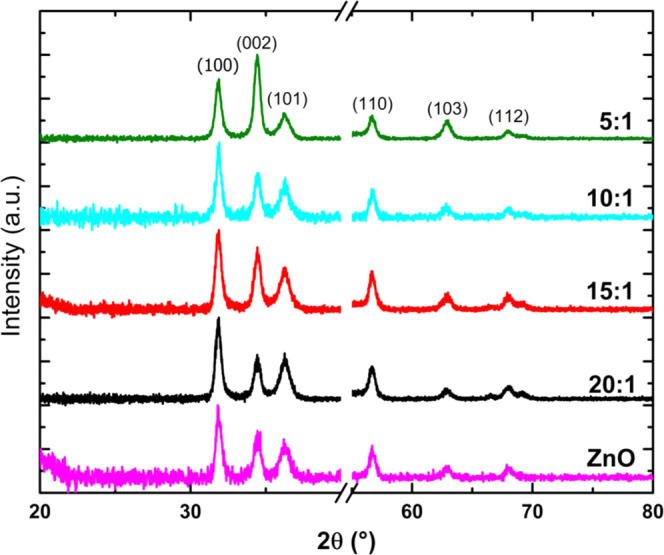
XRD patterns of the as-prepared undoped ZnO and ZnO/Zr films grown on SiO_2_/Si(100) substrates.

Figure 2 shows the high-resolution X-ray photoelectron spectra (XPS) for undoped and Zr-doped samples. Zn 2p, O 1s, and Zr 3d are identified, from which the quantification was carried out obtaining the following concentration values of Zr: 1.9, 2.06, 2.73, and 4.4 Zr atom % for ZnO–Zr ALD ratios of 20:1, 15:1, 10:1, and 5:1, respectively. X-ray diffraction patterns of the undoped ZnO and ZnO/Zr films grown on SiO_2_/Si(100) are summarized in Figure 3 corresponding to the wurtzite phase of ZnO (JCPDS #00-036-1451). The cell parameters of samples are listed in Table 1 and were calculated using the following equation^31^
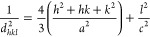
2

**Table 1:** Percentage of Zr Calculated from the XPS Spectra and Structural Parameters Deduced from the X-ray Diffraction Patterns of ZnO and Zr/ZnO Thin Films

sample	Zr (atom %)	cell parameters *a* = *b* (Å) ± 0.00032	cell parameter *c* (Å) ± 0.00009	crystallite size (nm)
ZnO	0	3.23101	5.18570	11.7
ZnO/Zr (20:1)	1.9	3.24176	5.20342	11.2
ZnO/Zr (15:1)	2.062	3.24123	5.20254	11.1
ZnO/Zr (10:1)	2.73	3.23911	5.20204	11.8
ZnO/Zr (5:1)	4.4	3.24174	5.20532	11.1

These results show that the (100) diffraction peak dominated in intensity on XRD patterns of all samples except the ZnO/Zr (5:1) sample, which exhibited higher intensity on the (002) peak, revealing that Zr impurification at very high concentrations modifies the preferential growth of ZnO/Zr films from the *a*-direction to the *c*-direction. Table 1 also indicates that Zr impurification slightly increases the cell parameter *c* of the wurtzite structure without exhibiting variations by increasing Zr concentration in samples. Furthermore, according to the literature, for films synthesized by ALD, the growth temperature, not the substrate atomic plane, defines its crystalline orientation because the ALD technique operates at higher deposition rates than required to generate an epitaxial growth.^32,33^ In this work, all syntheses were carried out at the same growth temperature and with the same doses to maintain the same growth rate, so we inferred that there are no differences between the three substrates used.

**Figure 4:**
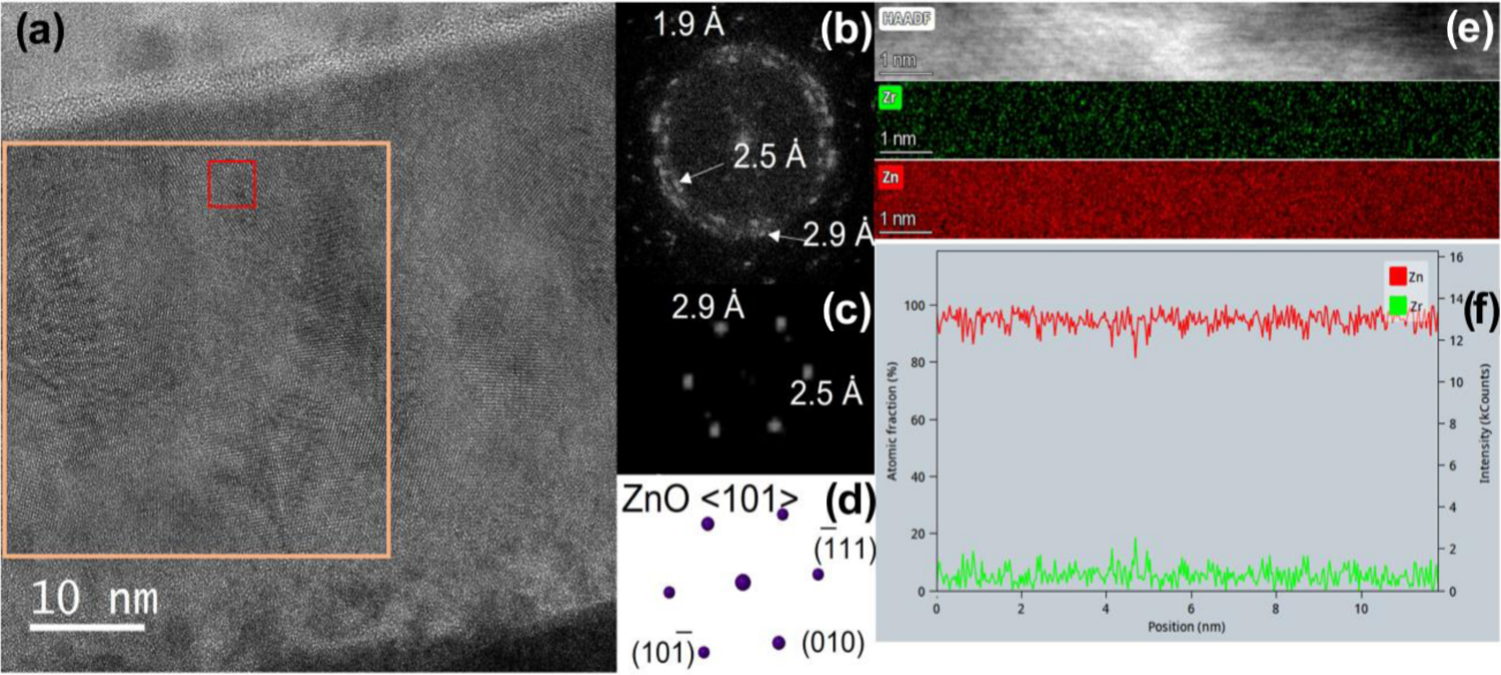
HREM image of the Zr–Zn layer (a), DDP of the orange (b) and red squares (c), simulated diffraction of the ZnO along the ⟨101⟩ axis zone (d), HAADF, energy-dispersive spectrometry (EDS) Zn and Zr (e) of the selected area, and Zn/Zr profile of the white line included in the HAADF image (f).

Figure 4a shows a representative HREM image of the ZnO/Zr 10–1 sample. The digital diffraction pattern (DDP) of a large area of the film generates a pattern like the one included in Figure 4b. We can observe several spots corresponding to spacings of 1.9, 2.5, and 2.9 Å, indicating that the analyzed area includes several nanodomains with slightly different orientations, but all of them in the axis zone ⟨101⟩ of ZnO. In fact, when the selected area is smaller (red square), the DDP can be identified as a single crystal of ZnO along ⟨101⟩ (Figure 4c,d).

To gain information on the distribution of Zr cations in the film, EDS–STEM experiments were carried out and are included in Figure 4e. We can observe that Zr atoms are homogeneously distributed in all of the regions. In fact, the line profile performed (Figure 4f) on the sample along the growth direction of the film shows a constant concentration for both Zn and Zr.

### 3.2 Construction of the Optical Model

**Figure 5:**
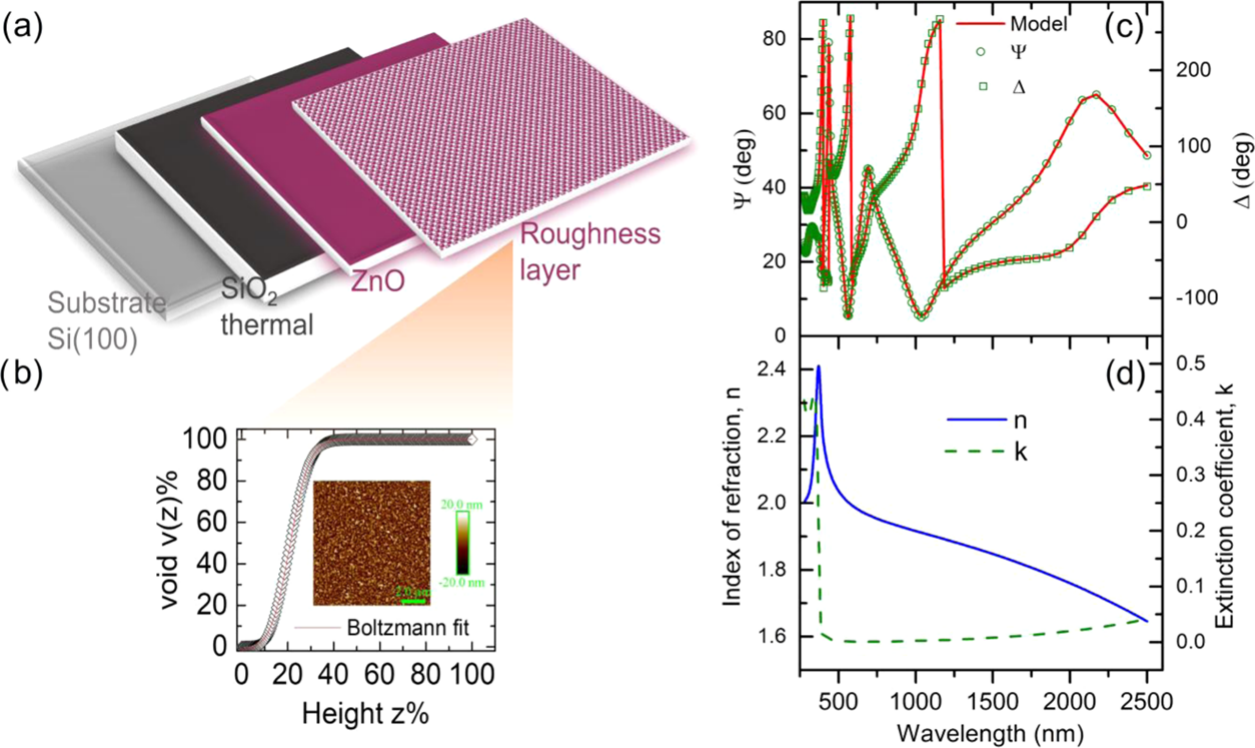
(a) Component layers of the optical model constructed for ellipsometry analysis of ZnO films. (b) Cumulative high distribution of the undoped ZnO film measured by AFM showing an accurate fitting with the sigmoidal Boltzmann equation. (c) Experimental ellipsometric amplitude ratio, Ψ, and phase difference angle, Δ, of the undoped ZnO film grown on SiO_2_/Si(100), and TL–Drude model fits (solid lines). (d) Refractive index, *n*, and extinction coefficient, *k*, for the undoped ZnO film deduced from the TL–Drude optical model.

The optical model used for spectroscopy ellipsometry (SE) measurements consisted of the ZnO/SiO_2_/Si(100) array shown in Figure 5a, composed of a roughness layer with thickness *t*_A_ present onto the surface of a ZnO film, with thickness *t*_Z_, grown on a SiO_2_ layer with thickness *t*_0_ = 300 nm and a silicon substrate with infinite thickness. The roughness layer, modeled as a ZnO/void ratio, was determined from the AFM topography images by fitting the cumulative height distribution as a function of the height to the sigmoidal Boltzmann equation
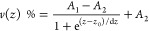
3to obtain the fitting parameters *A*_1_, *A*_2_, *z*_0_, and d*z* as shown in Figure 5b. Here, *v*(*z*) % represents the percentage of voids as a function of height, expressed in turn as a percentage of the surface roughness layer thickness, *z* %, *v*(*z* = 0) % being 0% at the ZnO bulk layer.^27^

The optical model parameters were calculated by fitting the dielectric function of the ZnO bulk layer, composed of two terms that correspond to two separate absorption processes: interband optical transition and free carrier absorption (FCA), observed in ZnO films with free carrier concentrations (*N*) relatively high (typically *N*_f_ > 10^18^ cm^–3^).^20^ Under such conditions of high doping level, the ZnO Fermi energy (*E*_F_) exhibits an upward shift toward the conduction band, filling it with free electrons and generating a semiconductor–metal transition. Due to this partially electron-occupied conduction band, the optical transition from the valence to the conduction band in such semiconductors does not occur at energies close to their band gap energies (*E*_g_), behaving then as a quasi-transparent material. Furthermore, since interband optical transitions occur between unoccupied conduction band states with energies higher than *E*_F_, the photon energy required to generate such interband transitions (*E*_inter_) becomes higher than *E*_g_. This effect observed in degenerate semiconductors is known as the Burstein–Moss shift.^20,33^ In contrast, FCA processes in semiconductors with high carrier concentration increase at longer wavelengths (or lower energies), and the extinction coefficient, *k*, increases significantly in this region. Therefore, the ZnO layer dielectric function can be modeled by combining the Drude, ε_D_, and the Tauc–Lorentz (TL), ε_TL_, oscillators to represent the FCA and the interband transitions, respectively.
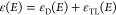
4

When the Drude oscillator is combined with other models, ε_D_ is described by
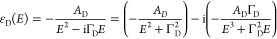
5where *A*_D_ and *G*_D_ are the amplitude and broadening parameters, respectively.

The TL model combines the empirical Tauc expression for the band edge onset with the absorption around band gap energy given by the classical Lorentz oscillator.^34^ This dielectric function is expressed with five free parameters: amplitude parameter (*A*), broadening parameter (*C*), TL optical gap (*E*_g_), peak transition energy (*E*_(*n*_0_)_), and energy-independent contribution to ε_1_(*E*) [ε_1∞_].
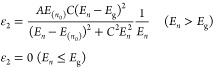
6

**Figure 6:**
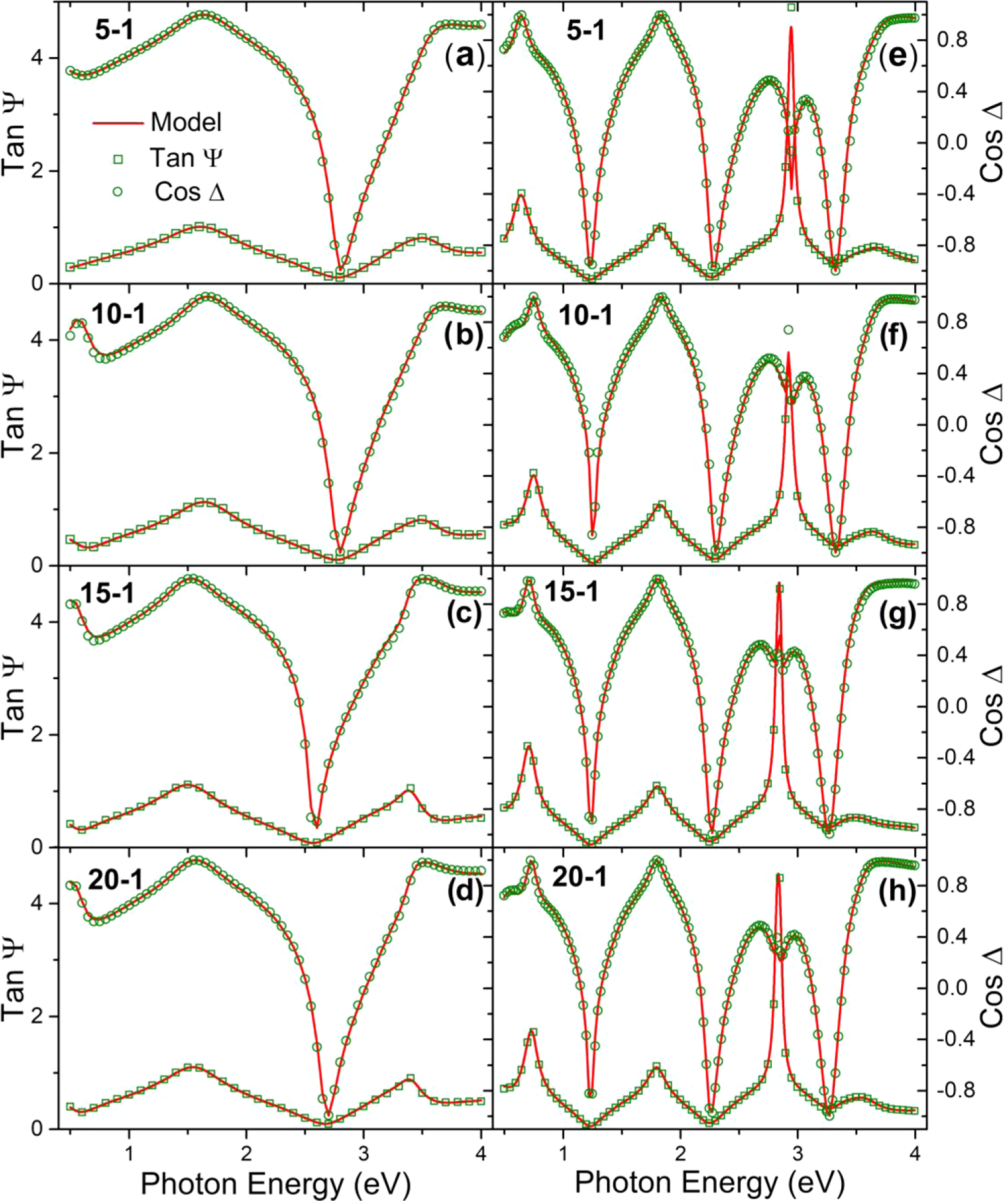
Experimental ellipsometric parameters, Ψ and Δ, of the as-prepared ZnO–Zr SiO_2_/Si(100) (a–d) and Si(100) substrate (e–h) films. The solid lines represent the fitting results from the TL–Drude analysis performed.

The expression for the real part, ε_1_(*E*), can be derived using the Kramers–Kronig relationship given that ε_1_(*E*) and ε_2_(*E*) (*n* and *k*) must be Kramers–Kronig consistent. The fitting procedure to determine the unknown optical model parameters was: (i) ZnO bulk layer thickness is first extracted for the optically transparent region (500–800 nm) followed by (ii) a point-by-point fit to generate the initial spectra in all measured regions, and finally, (iii) fitting of TL and Drude oscillator models to the final spectra and regression between the generated and measured data is performed. Therefore, a Levenberg–Marquardt regression algorithm was used to find the optical model parameters fitting the SE data until a minimum value of the mean-squared error (MSE) was obtained. The validity of the model was confirmed by the good values of MSE shown in the fittings (MSE < 5). This exact procedure was applied for the doped samples; the corresponding adjustments are shown in Figure 6.

### 3.3 Characterization of Optical Properties

Figure 5c displays the SE spectra of the undoped ZnO film grown on SiO_2_/Si(100) with the ellipsometric angles Ψ and Δ obtained at wavelengths between 350 and 2500 nm, at an incidence angle of 75°, besides their accurate fitting achieved with the TL–Drude model. Table 2 shows the values of the ellipsometry parameter calculated with this optical model. The refractive index, *n*, and extinction coefficient, *k*, are shown in Figure 3d, which exhibit for the undoped ZnO films a typical dispersion behavior for wavelengths higher than 370 nm. Furthermore, the high values observed for the refractive index, between 1.6 and 2.4, correspond with the good crystalline quality of the film. Figure 5d also shows the extinction coefficient of the film, revealing a sharp absorption peak below 420 nm associated with interband processes, besides a transparent region between 420 and 750 nm with a slight increase of *k* to higher wavelengths attributed to free-electron transitions.^32^

**Table 2:** Ellipsometry Parameters of the Undoped ZnO/SiO_2_/Si(100) Film Calculated Using the TL–Drude Model

ε_1_(∞)	3.60 ± 0.01
*A* (eV)	42 ± 2
*E*_(*n*_0_)_ (eV)	3.44 ± 0.004
*C* (eV)	0.172 ± 0.008
*E*_g_ (eV)	2.95 ± 0.01
*A*_D_ (eV)	5.87 ± 0.52
Γ_D_ (eV)	0.0541 ± 0.005
*t* (nm)	99.8 ± 0.3

**Figure 7:**
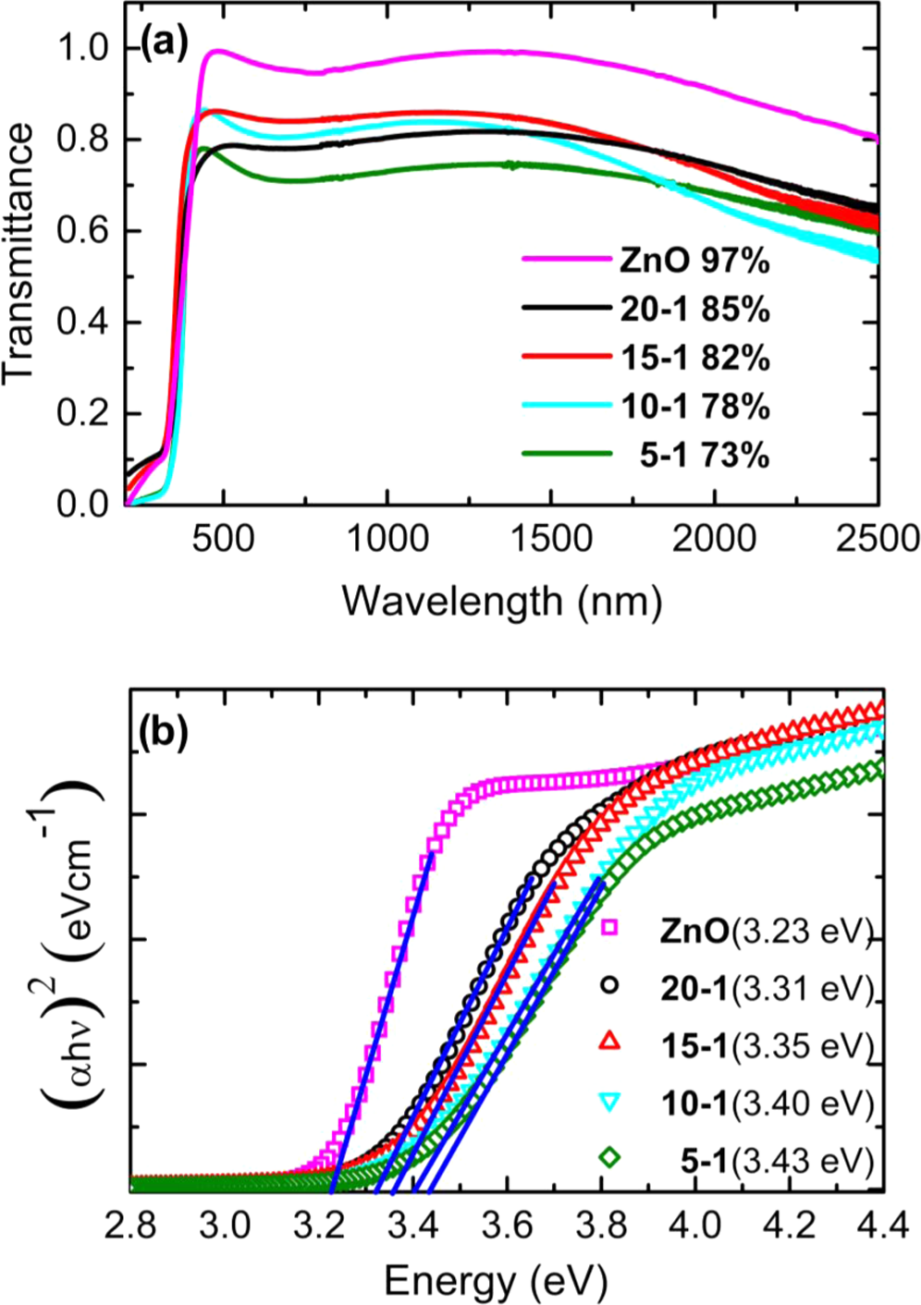
(a) Normal incidence optical transmittance of samples grown on sapphire and (b) α^2^ (deduced by ellipsometry model of samples grown on Si) versus *h*ν plots, fitted to a sigmoidal Boltzmann function to calculate the band gap, *E*_g_, whose values appear in parentheses for each sample.

Figure 7a shows the normal incidence optical transmittance spectra measured from ZnO/Zr films grown on sapphire substrates by UV–vis, which exhibit three different regions: (i) a high-absorption region (200 nm ≤ λ ≤ 450 nm) related with electronic band gap transitions, (ii) a high-transparency region in the visible range (400 nm ≤ λ ≤ 900 nm) with *T* values between 73 and 97% of external transmission (transmission can increase with proper coating), and (iii) an absorptive region in the NIR (900 nm ≤ λ ≤ 2500 nm) generated by the presence of free carriers in the films. The absorption coefficient of a crystalline semiconductor is expected to behave as^35^
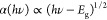
7where α is the absorption coefficient calculated from the ellipsometric model of the samples grown of Si and *h*ν is the photon energy. Thus, the band gap energy *E*_g_ is calculated by fitting the curves α^2^–*h*ν with a Boltzmann sigmoidal function using nonlinear regression (Figure 7b) and calculating the crossing point between the tangent line at the inflection point of the sigmoidal function and its lower asymptote.^36,37^ Legends in Figure 7b show the *E*_g_ values thus calculated for ZnO and ZnO/Zr films, revealing that this parameter increases with Zr doping until a maximum value of 3.43 eV is reached for the sample ZnO/Zr (5:1).

**Figure 8:**
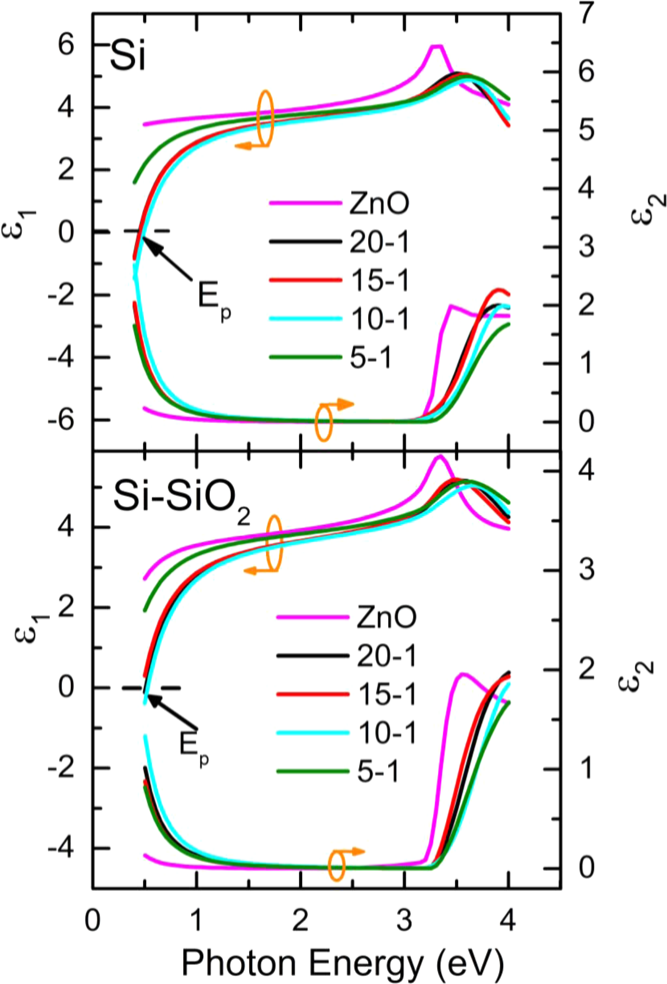
Real and imaginary parts of the dielectric function obtained from the optical model, plotted versus photon energy for the different samples deposited on Si (top) and Si–SiO_2_ (bottom).

The curves of the complex dielectric function, ε_1_, and ε_2_, as a function of the photon energy, of the undoped ZnO and ZnO/Zr films synthesized on Si(100), and SiO_2_/Si(100) substrates are shown in Figure 8a,b, respectively. The dielectric function of the undoped ZnO films shows similar behavior to that shown in previous reports^21^ (see Figure S2 in the Supporting Information). The peak of the real part ε_1_ that corresponds to free carrier and interband absorption appears at almost the same position and broadens due to the thickness when the film is compared to the bulk sample. The curves of Figure 8 reveal two relevant features: (i) both ε_1_ and ε_2_ peaks at *E* > 3.5 eV exhibit a slight shift toward higher energies with Zr doping; (ii) at lower energies, ε_2_ increases following the series of the ZnO/Zr films: 5:1, 20:1, 15:1, and 10:1, that is, it does not increase with Zr doping, while ε_1_ decreases in the same order. In addition, the plasma energy value *E*_p_, obtained from ε_1_ = 0, exhibits a slight increase accordingly with this sequence, indicating that samples are becoming increasingly electrically conductive. Similar behavior has been reported for Ga-doped ZnO thin films.^20^

### 3.4 Characterization of Electrical Properties

**Figure 9:**
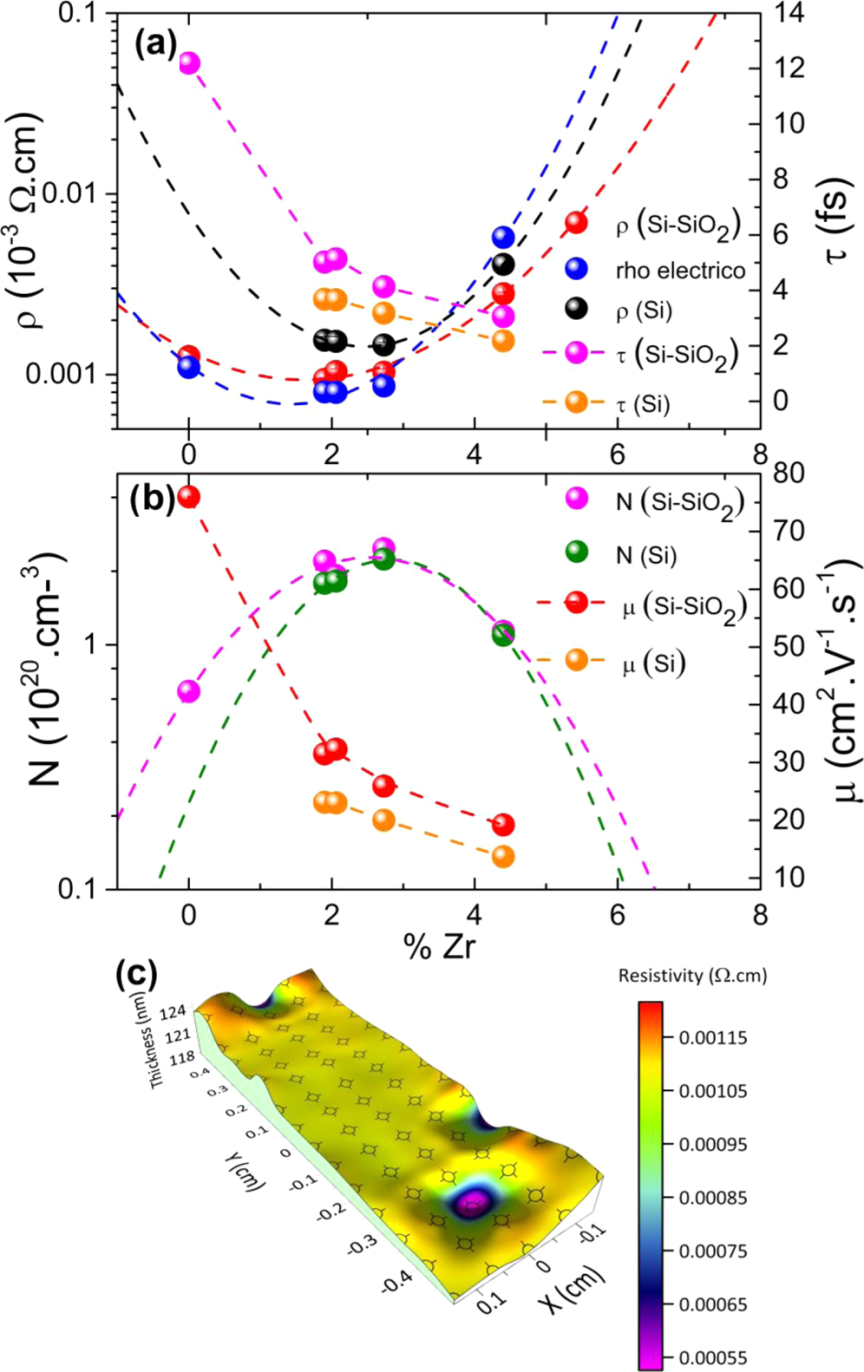
(a, b) Variation of ρ, τ, *N*_opt_, and μ with Zr atom %. Dashed lines are a guide to the eye. (c) SE mapping was performed over a wide (1 × 0.8 cm^2^) region of the 20–1 sample (1.9 atom % Zr).

To investigate the electrical properties of the undoped ZnO and ZnO/Zr films synthesized on Si(100), and SiO_2_/Si(100) substrates deduced from the optical measurements, the free carrier concentration *N*_opt_, resistivity ρ, mobility μ, and relaxation times τ were obtained by applying the classical Drude model expressions^38^
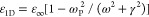
8
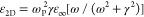
9where ω_P_ is the screened plasma resonance frequency given by
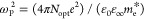
10where ε_∞_ and ε_0_ are the dielectric constants of the medium and free space, respectively, and *m*_e_^*^ is the effective mass of the charge carriers. Furthermore, *N*_opt_, ρ, and μ, τ exhibit the following relationships: 1/τ = −e/(*m*_e_^*^μ)] and ρ = 1/(*N*_opt_ e μ). Figure 9a displays the resistivity and relaxation time for the different Zr concentrations of analyzed ZnO/Zr films, revealing for those grown on SiO_2_/Si(100) a decrease in their resistivity from 1.7 × 10^–3^ Ω cm, observed for undoped ZnO, to 1.0 × 10^–3^ Ω cm in Zr-doped films with concentrations lower than 3 atom %, besides an increase to 2.8 × 10^–3^ Ω cm in the ZnO/Zr film with Zr concentration of 4.4 atom %. Figure 9b shows the carrier concentration and mobility values with the Zr concentration in samples. Accordingly, *N*_opt_ exhibits a maximum value at 2.73 atom % both for ZnO/Zr films grown on Si(100) and SiO_2_/Si(100) substrates, in agreement with the results reported by Ye et al.^100^ Then, it decreases since no more extra free electrons are now generated when Zn^2+^ is substituted by Zr^4+^ impurities, i.e., when the overdoping-like behavior appears.^14,15^

On the other hand, the mobility decreases from 76.0 to 19.2 cm^2^/(V s), where the Zr atom % corresponds to 0 and 4.4, mainly due to the increase of carrier scattering and the formation of Zr^4+^ impurities. For the interpretation of the ZnO Zr doping efficiencies, Figure 9c shows the results of the SE mapping performed over a wide (1 × 0.8 cm^2^) region of the ZnO/Zr (20:1) sample (1.9 Zr atom %). Here, the optoelectronic properties can be nondestructively characterized, and it allows modeling the resistivity within the layer while providing information about the thickness homogeneity. For example, it may be seen that the ZnO/Zr (20:1) film with an average thickness of 119 nm shows a thickness variation of 5% with lower ρ values in the thinner regions.

**Figure 10:**
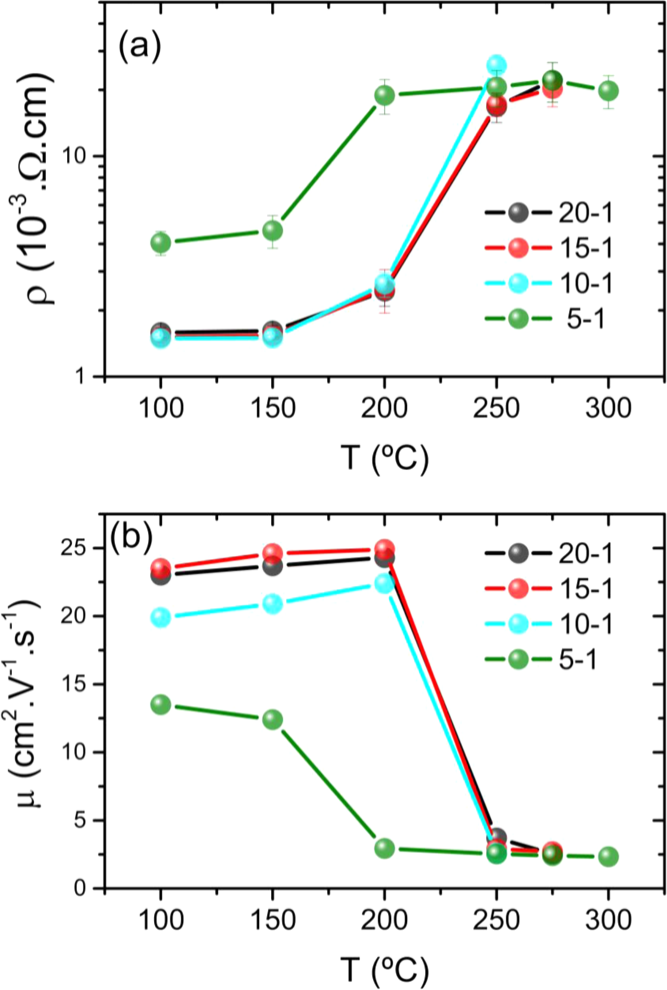
Variation of (a) resistivity and (b) carrier mobility deduced for the different Zr-doped ZnO films as a function of annealing temperature.

Figure 10a shows the resistivity values of the ZnO/Zr films synthesized on Si(100) substrates measured at different annealing temperatures, revealing that the resistivity of films increases by increasing annealing temperature until it reaches a maximum value at *T*_s_, the saturation temperature. This effect is mainly produced because the annealing treatment enhances the oxygen atom content in the films, which is absorbed by the interstitials Zn-rich surface to form insulator ZnO molecules. Consequently, the number of native oxygen vacancies acting as donors in ZnO decreases, making the film more resistive. At *T*_s_, most of the oxygen atoms are absorbed and resistivity remains constant.^39,40^ Interestingly, all of the studied Zr-doped ZnO films saturate at the same resistivity value of 2 × 10^–2^ Ω cm. Furthermore, the electron mobility exhibited a substantial decrease in ZnO/Zr films thermally treated at temperatures higher than 250 °C (Figure 10b), which we attribute to the fact that the mobility of free carriers decreases to a low limit, not decreasing below it with increasing the annealing temperature, favoring the elimination of ZnO native point defects, as Zn interstitials and oxygen vacancies.

**Figure 11:**
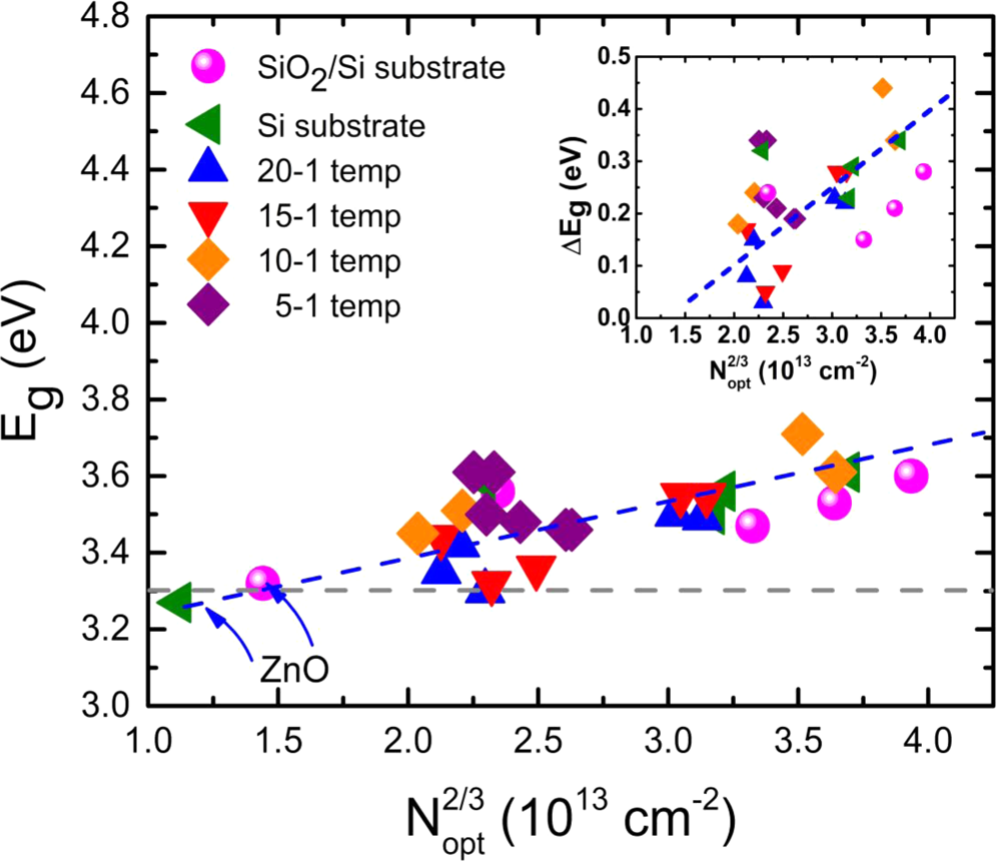
Plot of the band gap energy *E*_g_, obtained from the Tauc plots by fitting a Boltzmann sigmoidal function in Figure 7, for all of the samples studied, represented as a function of *N*_opt_^2/3^. The inset shows Δ*E*_g_ in accordance with the Burstein–Moss equation. As a reference, the horizontal dash-dotted gray line corresponds to the band gap of single-crystal ZnO (3.3 eV), according to Srikant and Clarke.^42^

Finally, Figure 11 displays a graph with *E*_g_ values as a function of *N*_opt_^2/3^ for all of the different undoped ZnO and ZnO/Zr/SiO_2_/Si(100) studied films, both as-prepared and annealed ones, which reveals that the absorption edge of all Zr-doped ZnO films shifts toward high photon energies, regardless of the type of substrate and annealing treatment applied. This *E*_g_ widening has its origin in the fact that the high electron concentrations involved in the absorption processes cause the Fermi-level position to move upward due to the increase of free electrons causing the absorption axis to shift toward higher photon energies, i.e., *E*_g_ is blue-shifted. This behavior is described as the effect of Burstein–Moss (BM), which is expressed by
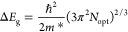
11where ℏ is the reduced Planck constant (ℏ = *h*/2π) and m* is the electron effective mass. Although band gap widening by BM effect can be calculated from eq 11, the actual shift of *E*_g_ – Δ*E*_g_, does not follow this equation, as it can be observed in the inset of Figure 11, where the dispersion of the experimental points around the tendency line is very large compared to that observed in the main graph of Figure 11. In addition to the BM effect, a band gap narrowing (BGN) effect can also exist in doped semiconductors, which has been explained by many-body effect of free carriers on the conduction and valence bands, known as band gap renormalization.^41^ It is well known that in heavily doped semiconductors, band gap widening and narrowing can exist simultaneously, with BGN being competitive with the BM shift. Therefore, it can be stated that the Zr-doped ZnO samples in this work present a band gap shift that is affected by both BM and BGN effects.

## 4 Conclusions

In this study, we have prepared ZnO and Zr-doped ZnO films of thickness ∼100 nm on sapphire, SiO_2_/Si(100), and Si(100) substrates using the ALD technique. The optical transmission of the films is good, with transmittances higher than 70% in the visible range for all cases and reaching a maximum of 85% for the 20–1 sample. We employed Drude–TL oscillators to model the films’ optical response and their optical constants. The absorption observed at the NIR region indicates a metallic behavior due to the presence of free carriers to 2.73 atom % doping. Furthermore, resistivity and carrier concentration exhibit a parabolic dependence with Zr concentration. Both yield minimum and maximum values, respectively, at the same critical value of 2.73 atom % Zr. Meanwhile, annealing temperature decreases the conductivity and carrier mobility of the films. Then, by controlling the doping level during the preparation of the films and annealing temperature during postprocessing, the electrical and optical properties of these Zr-doped ZnO thin films can be tuned.

Finally, our results show that the band gap energy follows the Burstein–Moss effect for all of the substrates and annealing temperatures. The relatively large stability of the main electro-optical parameters with annealing treatments demonstrates their applicability to develop TCOs to be used in high-power electronic devices.

## Supporting Information Available

Comparison of the dielectric function of ZnO films obtained in this work compared to that of bulk ZnO (from refs S1–S3) (PDF)
