# Supplementary material for: Spectroscopic Ellipsometry Study on Tuning the Electrical and Optical Properties of Zr-Doped ZnO Thin Films Grown by Atomic Layer Deposition
Source: ACS Appl Electron Mater. 2022 Feb 24;4(3):925–35. doi: 10.1021/acsaelm.1c01026 (PMC9121516; doi:10.1021/acsaelm.1c01026)
Supplement: Supplementary file 1 — el1c01026_si_001.pdf [file el1c01026_si_001.pdf]

A spectroscopic ellipsometry study on tuning electrical and optical properties in Zr-doped ZnO thin films grown by atomic layer deposition

*Carolina Bohórquez [1], Hicham Bakkali [2,3], Juan J. Delgado [3,4], Eduardo Blanco [2,3], Manuel Herrera [5], and Manuel Domínguez [2,3]**

[1] Centro de Investigación Científica y de Educación Superior de Ensenada (CICESE), 22860 Baja California, Mexico.

[2] Departamento de Física de la Materia Condensada, Campus de Puerto Real, Universidad de Cádiz, E11519 Puerto Real, Spain.

[3] Institute of Research on Electron Microscopy and Materials (IMEYMAT), Campus de Puerto Real, Universidad de Cádiz, E11519 Puerto Real, Spain.

[4] Departamento Ciencias de los Materiales e Ingeniería Metalúrgica y Química Inorgánica, Campus de Puerto Real, Universidad de Cádiz, E11519 Puerto Real, Spain

[5] Centro de Nanociencias y Nanotecnología, Universidad Nacional Autónoma de México Ensenada, Baja California, Mexico

*Email: manolo.dominguez@uca.es

Supporting Information

The real part of the dielectric function is shown above in the Figure S1 and compares two spectra of bulk ZnO with our samples. In all cases, the transparent region has the same shape. In the absorption region, the peak with the lowest energy is the Adachi, 1999[1] peak at 3.2 eV, followed by Shih, 2009[2] spectrum with a sharper peak at 3.31 eV and our thin-film ZnO with the peaks at 3.31 eV for the sample with Si substrate and 3.36 eV for the ZnO grown over SiO_2.

The bottom of the Figure S1 shows the imaginary part of the dielectric function; the optical response occurs between 3.2 eV and 3.3 eV for the bulk ZnO, representing the threshold value for the direct optical transition from VB to CB. In comparison, the samples ZnO ALD (grown over SiO2 and Si) have a less sharp transition starting at 3.2 eV and ending at 3.4 eV. This broadening of the absorbance peak has been attributed to the decrease in film thickness.

On the other hand, Adachi's work does not consider the roughness layer, which in our work is taken from the measurements made by AFM.

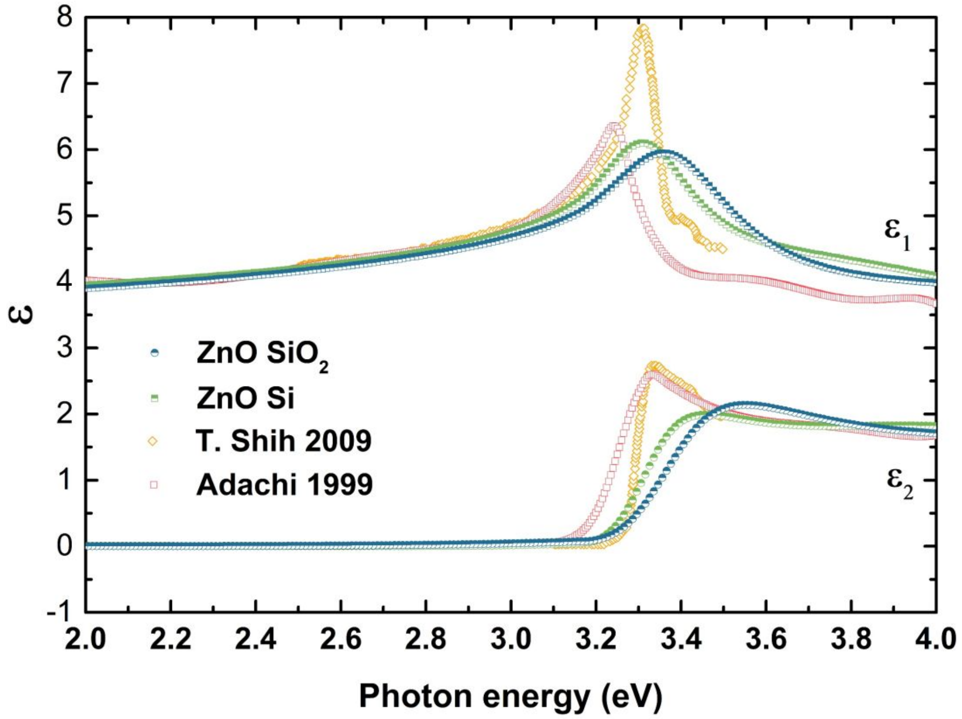

Figure S1. Real and imaginary dielectric function of bulk ZnO compares with un-doped ZnO thin films from this work.

As mentioned in the introductory part of the article, a crucial parameter is the thickness of the films. In Figure S2. Our undoped ZnO film is compared again with the dielectric function of bulk ZnO from Samarasingha's work[3], where they also study ZnO thin films made by ALD. We see that our film follows the tendency shown there. The peak appers at almost the same photon energy and is broadened due to the thickness, when the film is compared to the bulk ZnO.

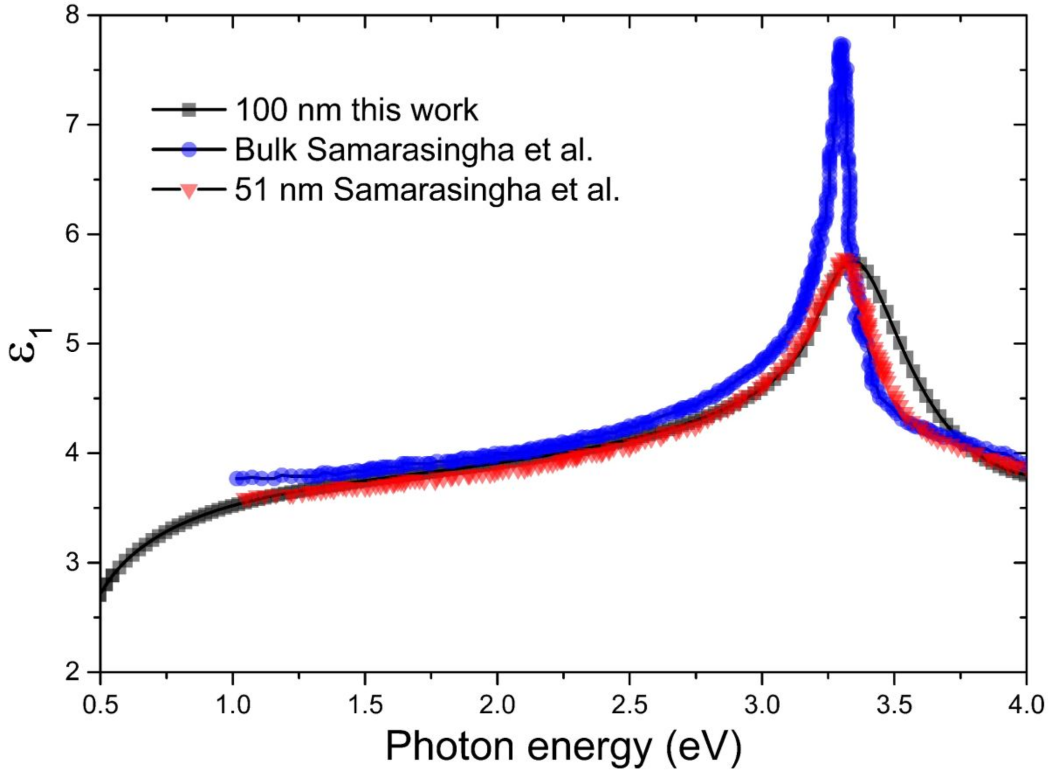

Figure S2. The figure shows a comparison between the real part of the dielectric function obtained in our work ALD thin film ZnO 100 nm, black, bulk ZnO blue, and the ALD thin film of thickness 51 nm red from Samarasingha[3].

References

(1) Adachi, S. Optical Constants of Crystalline and Amorphous Semiconductors. Optical Constants of Crystalline and Amorphous Semiconductors 1999. https://doi.org/10.1007/978-1-4615-5247-5.

(2) Shih, T.; Winkler, M. T.; Voss, T.; Mazur, E. Dielectric Function Dynamics during Femtosecond Laser Excitation of Bulk ZnO. Applied Physics A 2009 96:2 2009, 96 (2), 363–367. https://doi.org/10.1007/S00339-009-5196-0.

(3) Samarasingha, N. S.; Zollner, S.; Pal, D.; Singh, R.; Chattopadhyay, S. Thickness Dependence of Infrared Lattice Absorption and Excitonic Absorption in ZnO Layers

on Si and SiO 2 Grown by Atomic Layer Deposition . Journal of Vacuum Science & Technology B 2020, 38 (4), 042201. https://doi.org/10.1116/6.0000184.
